# Immediate Effects of the Mandibular Muscle Energy Technique in Adults with Chronic Temporomandibular Disorder

**DOI:** 10.3390/clinpract14060202

**Published:** 2024-11-25

**Authors:** Antonio Márquez-Vera, Luis Polo-Ferrero, Ana Silvia Puente-González, Roberto Méndez-Sánchez, José Antonio Blanco-Rueda

**Affiliations:** 1 Department of Nursing and Physiotherapy, University of Salamanca, 37007 Salamanca, Spain; amarvefisio@usal.es (A.M.-V.); pfluis@usal.es (L.P.-F.); silviapugo@usal.es (A.S.P.-G.); 2 Institute for Biomedical Research of Salamanca (IBSAL), University Hospital of Salamanca, 37007 Salamanca, Spain; jablanco@saludcastillayleon.es; 3 Maxillofacial Surgery Department, University Hospital of Salamanca, 37007 Salamanca, Spain; 4 Department of Medicine, University of Salamanca, 37007 Salamanca, Spain

**Keywords:** temporomandibular disorder, temporomandibular joint, manual therapy, non-surgical management, orofacial rehabilitation

## Abstract

Background/Objectives: Temporomandibular disorders affect the muscles used for chewing, the temporomandibular joint, and other related tissues, resulting in pain, limited mobility, and dysfunction of the masticatory muscles. Physical therapy plays a critical role in treatment. Manual therapy can trigger neurophysiological mechanisms that contribute to pain relief and a reduction in muscle activation. Evaluations of different manual therapy techniques are needed on this topic. The main objective of this study was to evaluate the effects of a specific manual therapy technique (the mandibular muscle energy technique) in adults with temporomandibular disorders. Methods: A randomized, parallel clinical trial was conducted, and 31 participants were recruited into an experimental group and 30 were recruited into a control group in order to analyze its effects on outcomes such as pain, pain threshold to pressure, mandibular mobility, and kinesiophobia. Pre- and post-intervention assessments were performed, followed by statistical analyses to verify the intra- and intergroup changes. Results: The results showed that the mandibular muscle energy technique produced positive effects with significant differences in the intra- and intergroup comparisons for pain threshold to pressure, mandibular mobility, and kinesiophobia, demonstrating its efficacy and safety as a treatment option for adults with temporomandibular disorders, with proven effects in the short term. Conclusions: The effects obtained and the absence of side effects showed that this technique can be integrated into multimodal treatment along with other types of interventions in patients with temporomandibular disorders.

## 1. Introduction

Temporomandibular disorders (TMDs) are heterogeneous musculoskeletal conditions affecting the masticatory muscles, the temporomandibular joint (TMJ), and related structures. They are the most common non-odontogenic source of orofacial pain and rank as the second most frequent musculoskeletal conditions after back pain [1]. Classically, these conditions are characterized by pain, limited mobility, and dysfunction of the masticatory muscles, with pain being the main reason for medical consultation [2,3]. The prevalence of TMDs in the European population is 29% [4]. Most studies have focused on evaluating pain-related interventions, as identified in a retrospective study of 4528 TMD patients, where orofacial pain, ear discomfort, and tenderness of the TMJ were common manifestations [5,6].

The OPPERA (Orofacial Pain: Prospective Evaluation and Risk Assessment) study revealed that out of a group of 2737 adults aged 18–44 years old in the United States, 260 developed initial TMDs during a follow-up period of 2.8 years, indicating an annual incidence of 4% [2]. TMDs represent a significant clinical challenge due to their multifactorial nature and complex etiology, which is often not fully understood. These disorders, sometimes of unknown origin, result from a complex interplay of biological, environmental, social, emotional, and cognitive factors, with a wide range of predisposing, initiating, and perpetuating factors [7]. Since 1992, there has been a shift in focus to a more comprehensive understanding of TMDs, considering not only clinical but also social, emotional, and cognitive aspects [8]. This evolution was supported by an ongoing review process, culminating in 2014 with an update to the diagnostic and treatment approaches that remains relevant to current clinical practice [1].

The relationship between degenerative changes in the TMJ and pain remains a matter of debate, although some studies suggest a correlation. However, the association of TMJ pain with an unfavorable prognosis in TMD treatment highlights the importance of addressing this component in clinical management [9,10]. In addition, psychosocial factors such as depression and anxiety contribute significantly to TMD symptoms, underscoring the importance of a comprehensive assessment that considers both physical and psychosocial aspects [11,12]. Typical manifestations of TMD patients include the presence of myofascial trigger points (MTPs) in the neck and masticatory muscles, which are relevant to the pathophysiology and manifestations of TMDs [13]. These MTPs are identified as tender areas in tight bands of skeletal muscle or muscle fascia, and stimulating them can elicit various symptoms [14]. Myofascial pain is commonly found in about 42% of patients with TMDs, making it a frequent diagnosis in this population [15]. Myofascial pain syndrome not only affects the muscles involved in TMDs but also involves the trapezius and other cervical muscles and back muscles, too. This muscle tends to be overloaded in cases of TMDs associated with a forward head posture, which increases its activity compared to a normal position [16]. In terms of the treatment options for patients with TMDs, there are two main categories: conservative and invasive [17]. Conservative treatments for TMDs include medication, physiotherapy, occlusal splints, self-care strategies, and interventions based on cognitive–behavioral approaches, and these should be the first-line treatment options [18]. An appropriate therapeutic approach to the treatment of TMD should focus on alleviating the main signs and symptoms of this pathology, with special emphasis on pain reduction as the main objective [12]. Physiotherapy plays an important role in treatment, with techniques such as manual therapy, therapeutic exercise, and electrotherapy used [19]. Through manual therapy, it is possible to trigger neurophysiological mechanisms that contribute to pain relief and a reduction in muscle activation [20]. Non-invasive rehabilitation treatment options relieve pain in 40% to 90% of patients [21]. Despite the wealth of existing literature on manual therapy for TMDs, a recent systematic review highlighted the need for further research due to the variability, methodological limitations, inconclusive data, and lack of homogeneity in the studies available [22]. For these reasons, it is essential to continue researching and designing rigorous studies on different manual therapy techniques. In the present study, the hypothesis was to test whether the mandibular muscle energy technique (MMET) is effective in the treatment of TMDs. The aim of this study was to see whether this technique was able to reduce orofacial muscle hyperactivity, improve maximum mouth opening (MMO), and reduce pain in adults with TMD.

## 2. Materials and Methods

### 2.1. Study Design

A randomized clinical trial was conducted with two parallel groups: an experimental group (EG) and a control group (CG). The purpose of this study was to evaluate the immediate effect of the MMET, a specific manual therapy technique.

The reporting of this clinical trial complies with the CONSORT 2010 statement [23]. The trial protocol received approval from the Comité Ético de Investigación con Medicamentos del Área de Salud de Salamanca under code PI 2019 11 386 and was conducted in accordance with the Declaration of Helsinki [24]. The clinical trial was registered at clinicaltrials.gov with identification number NCT05594511.

In this study, a double-blind design was adopted to ensure methodological control, where both the participants and the evaluating researcher were unaware of whether the treatment received was the technique to be evaluated or the placebo. Thus, the participants did not know whether they were receiving the real intervention or a sham technique, and the evaluating researcher also had no information about the allocation of the participants into each group. Recruitment was carried out according to convenience as patients went to the doctor due to signs and symptoms. Randomization was performed using the spreadsheet program “Excel”, utilizing the random function with a 1:1 ratio.

### 2.2. The Study Population

**Figure 1:**
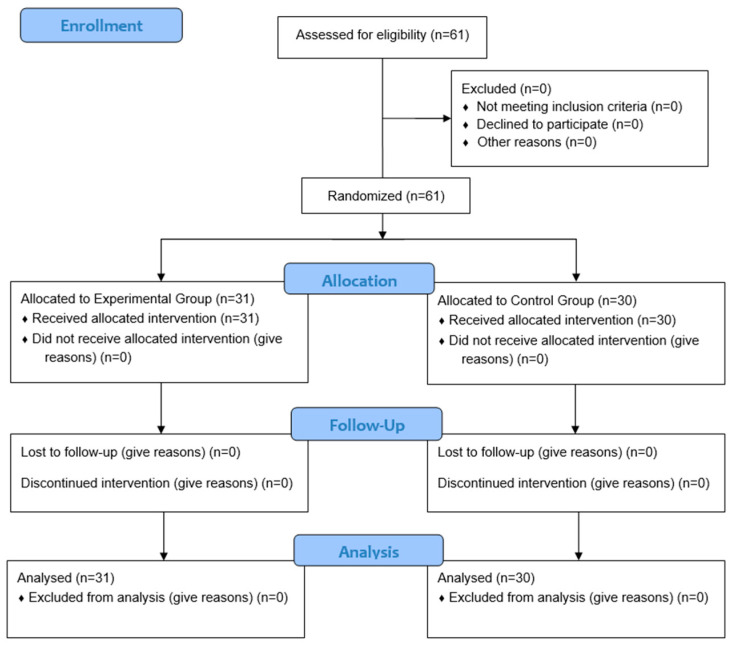
Flow diagram. CONSORT 2010.

Participants were recruited from the Maxillofacial Surgery Department of the University Hospital of Salamanca. They were then contacted by telephone to coordinate their attendance at the initial evaluation. Prior to their participation in the clinical trial, all the participants provided voluntary informed consent, which was read and signed. A total of 61 patients were recruited and assigned into the two groups, as shown in the flow chart (Figure 1).

This study included adults of both sexes, aged between 18 and 65 years old, who presented with painful TMJ symptomatology. TMDs are highly prevalent in adults, peaking between the ages of 20 and 40 and declining in later life [1]. This approach allowed the results to be extrapolated to a wider and more realistic population, making the findings applicable to a diverse range of adults with TMDs beyond a specific subgroup. In addition, the effectiveness of the test could be demonstrated, despite the variability present in the population aged 18–65 years old. These individuals had previously been diagnosed with TMD by a physician using the updated criteria and had experienced symptoms for more than 3 months, so the syndrome could be considered chronic [1]. This study included patients with both joint and muscle involvement. This study excluded adults with congenital malformations and patients who had received physiotherapy treatment in the month prior to the study or had ingested medication in the 8 h prior to the initial evaluation, as well as using any other circumstances that could have interfered with the purpose or development of the study as exclusion criteria at the discretion of the researchers.

### 2.3. Assessment

The evaluation took place at the Teaching and Assistance Unit of the Faculty of Nursing and Physiotherapy at the University of Salamanca, which is a fully equipped facility with optimal conditions. The initial and final evaluations were conducted during a single visit, with the treatment being applied between the two visits. Outcome variables were recorded at both visits, and sociodemographic and anthropometric data were collected during the first visit. Before the tests, each participant received an information sheet that outlined the objectives, methodology, and expected results. Verbal answers were provided to any questions, and participants were asked to sign an informed consent form.

During the evaluation, sociodemographic variables, including age, sex, height, weight, and body mass index (BMI), were collected from each participant. To measure pain, the Visual Analog Scale (VAS) was used, which is a tool that quantifies pain intensity. This study measured the pain threshold to pressure (PTP) using a PCE-FM 200 dynamometric algometer at the specific trigger points described by Travell and Simons, including MTP1 of the upper trapezius, MTP1 of the masseter, MTP1 of the lateral pterygoid, and MTP1 of the digastric muscle [14]. Additionally, the MMO was recorded using a vernier caliper that measured the opening and lateralizing movements of both sides in relation to the teeth. The measurements were averaged from three trials, with a 10 s rest interval between each measurement, alternating between sides. In addition, the kinesiophobia levels were evaluated using the TAMPA scale adapted for TMDs [25]. The assessment was conducted initially by the investigator and then by telephone for a second assessment after 7 days.

### 2.4. Interventions

Following the initial assessment and the assignment of the participants into their respective groups, the intervention was carried out in both the experimental and control groups. Only one intervention session was carried out (the MMET vs. the placebo), and the final assessment was then carried out.

**Figure 2:**
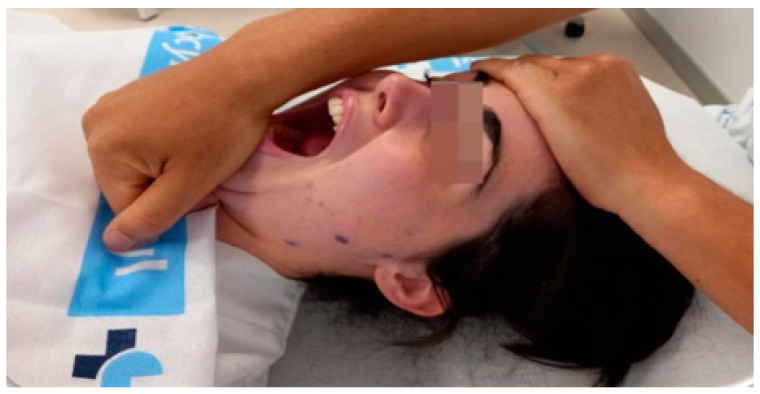
Image of the manual intervention process using the MMET.

The experimental group: A passive opening movement was performed to the limit of the range of motion (ROM), followed by isometric contractions of the closing muscles for 3 to 5 s, repeated in three cycles with rest periods between contractions. After each cycle, an attempt was made to improve opening mobility until a new motor barrier was encountered, followed by a waiting period before initiating the next cycle. The mandible was passively returned to the closed position at the end of the third cycle [26]. An image of the intervention process can be seen in Figure 2.

The control group: The participants underwent a simulated suboccipital muscle inhibition technique. as used in similar studies [27,28]. The physical therapist stood at the head of the patient’s table and positioned their hands under the participant’s skull, with their fingertips in contact with the occipital base for 5 min without applying pressure or having a therapeutic intent.

### 2.5. Sample Size Calculation

The sample size was calculated based on the primary study variable, the VAS, using GRANMO tool version 7.12 from April 2012. For patients with TMDs, the minimum clinically important difference in the VAS was estimated at 1.5 points [29]. An alpha risk of 0.05, a beta risk of 0.2 in bilateral contrast, and a loss rate of 5% were considered. It was concluded that a minimum of 30 subjects per group would be necessary to detect a difference of 1.5 units or more, assuming a standard deviation of 2 units.

### 2.6. Statistical Analysis

The data analysis was performed using IBM SPSS Statistics version 25.0. Descriptive statistics were presented as means and standard deviations for continuous variables and frequencies and percentages for categorical variables. Normality was assessed by observing normality plots and verified by the Shapiro–Wilk test to ensure the homogeneity of all of the variables. An analysis was conducted on the difference between the means of the two scores. The confidence level used was 95% (0.05).

For the inferential analysis of the hypothesis contrast, the effect of the intervention in the EG was assessed compared to the CG. An intragroup comparison analysis (pre–post) was used in each of the groups (using Student’s *t*-test for dependent samples as a parametric test or Wilcoxon’s test as a non-parametric test), in addition to an intergroup comparison at the end of the study of the post-intervention results for all outcome variables (Student’s *t*-test for independent samples as a parametric test or the Mann–Whitney test as a non-parametric test). In addition, the effect size was calculated using Cohen’s d to quantify the magnitude of the differences between groups. The interpretation of the effect size was based on Cohen’s criteria, considering it small (0.2–0.4), medium (0.5–0.7), or large (≥0.8). Correlations between variables were evaluated using Pearson’s or Spearman’s correlation coefficient, as appropriate, and were classified as weak (0.1–0.3), moderate (0.4–0.6), or strong (0.7–1.0), following Cohen’s classification [30].

## 3. Results

### 3.1. Participant Characteristics

This study comprised 61 participants, with 30 in the CG and 31 in the EG. The mean age of the CG was 38.47 ± 11.39 years, while that of the EG was 40.13 ± 10.28 years. All participants completed the study without any sample losses. No participant reported any adverse effects in any group. Female participants predominated, accounting for 81.96% of the total (*n* = 50), while men accounted for 18.04% (*n* = 11). It is important to note that homogeneity between the groups in terms of sex was maintained, with five men in the CG and six in the EG, showing no significant differences in the Shapiro–Wilk test (*p* = 0.785).

Analysis of the normality of the other variables indicated that in most of them, no significant differences were observed before the intervention (*p* > 0.05), except in the opening and left deductive ROM (*p* = 0.007 and *p* = 0.044, respectively). Inferential analysis of the latter two variables was performed using non-parametric tests.

The baseline values for each variable for both groups, as well as the analysis of normality, are detailed in Table 1.

### 3.2. Results of the Outcome Variables

Following the intervention, significant differences were observed between the two groups in all outcome variables (*p* < 0.001). The EG showed an improvement in all variables, while the CG either maintained their condition or worsened. Table 2 presents detailed values for each variable after the intervention for both groups, along with the corresponding inferential analysis.

In the EG, pain measured by the VAS showed a significant reduction (pre: 5.69 ± 1.95; post: 5.20 ± 1.81; *p* < 0.001), while in the CG, a slight increase was observed (pre: 5.44 ± 2.67; post: 5.58 ± 2.59; *p* = 0.013). Intergroup comparison showed a significant difference (*p* < 0.001), albeit with a small effect size (d = 0.427).

**Figure 3:**
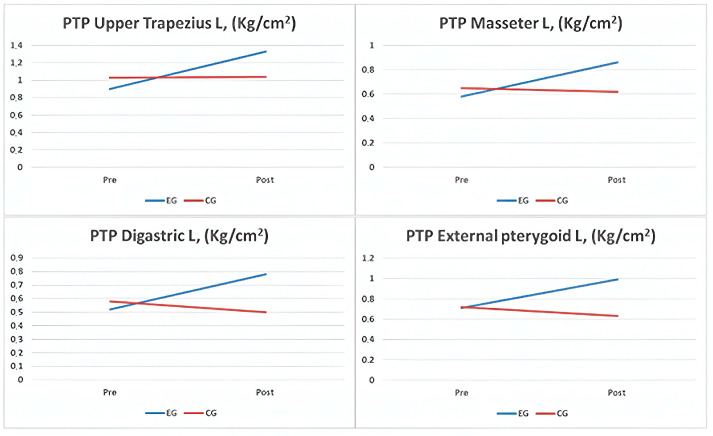
Graphs of comparison of results on left muscle pain thresholds to pressure in both study groups. Abbreviations: PTP: pain threshold to pressure; L: left; EG: experimental group; CG: control group; kg/cm^2^: kilograms/square centimeters.

For the PTP in various muscles, the EG exhibited significant improvements in the right trapezius (*p* = 0.025), left trapezius (*p* = 0.011), right masseter (*p* = 0.009), left masseter (*p* = 0.014), right external pterygoid (*p* = 0.012), left external pterygoid (*p* = 0.009), right digastric (*p* = 0.017), and left digastric (*p* = 0.013) muscles. However, in the CG, no significant changes were observed in these areas, except in the left trapezius (*p* = 0.047) and left digastric (*p* = 0.004) muscles, which were counterproductive (Figure 3). Intergroup comparisons showed significance in all areas (*p* < 0.001), with small effect sizes between 0.184 and 0.371.

The results show a significant increase in the ROM in the EG, with a median of 38.77 mm (IQR: 34.04–45.83) before and 50.49 mm (IQR: 46.27–51.87) after the intervention (*p* < 0.001), while the CG did not show significant changes (median: 33.39 mm; IQR: 23.87–39.25; *p* = 0.149). The difference between groups was significant (*p* < 0.001; d = 0.742). For right deviation, the EG showed a significant increase (*p* < 0.001), while the CG experienced no relevant changes (*p* = 0.275; d = 0.453). In left deviation, the EG increased in its ROM from 9.19 mm (IQR: 6.98–10.22) to 11.09 mm (IQR: 9.2–12.13; *p* < 0.001), with no changes in the CG (*p* = 0.242; d = 0.602). Kinesiophobia was reduced in the EG (pre: 30.32 ± 6.77; post: 23.58 ± 5.81; *p* < 0.001), while there was no significant change in the CG (*p* = 0.156). The difference between groups was significant (*p* < 0.001), although with a small effect size (d = 0.259).

With regard to the MMO, the largest statistically significant differences were found here (*p* < 0.001). The EG showed an increase of 8.58 mm (22%) in their opening movement. Additionally, both lateral deviations showed a 20% improvement, measuring 1.79 and 1.76 mm, respectively, while the CG’s values remained stable.

Furthermore, one week after performing the technique, an improvement in fear of jaw movement was observed. The EG participants recorded a decrease of 6.74 points on the TAMPA scale, whereas the CG experienced a decrease of only 0.84 points on the same scale.

## 4. Discussion

### 4.1. Discussion of the Results

This study’s findings support the MMET as an effective intervention for treating TMDs. Significant improvements were observed in pain relief, PTP, MMO, and reduced fear of movement in patients who received the MMET compared to those who did not. This study highlights the effectiveness and safety of the MMET as a viable therapeutic option for adults with TMDs. This is in contrast to other interventions that may have adverse effects, especially in the short term. Additionally, the MMET has been shown to have beneficial effects from the first moment it is administered.

This study also supports previous research findings that have demonstrated the effectiveness of the MMET in reducing pain and improving MMO. These positive effects were sustained for up to four weeks after the intervention, as previously observed in other studies [31,32]. However, it is important to consider a previous study that reported an increase in the number of bruxism episodes per hour of sleep in patients treated with the MMET, despite improvements in pain and MMO [29]. Interpretation of these results should be approached with caution due to the small sample size in said study. It is important to analyze whether the intensity of the technique used in this study was appropriate. The same results were also obtained with techniques similar to the MMET, such as myofascial release [33,34]. However, it is important to note that previous studies did not include measurement of PTP, as we did in our study. Including this variable is essential for obtaining a more complete assessment of a patient’s response to treatment. This objective measure may be particularly valuable in identifying changes in pain perception, complementing the subjective assessments made with the VAS. Since the VAS is a subjective measure, improvements in outcomes can be attributed, in part, to individual participants’ perception. The incorporation of objective measures provides a more robust and multidimensional assessment of pain, allowing for a more accurate interpretation of the treatment effects and reducing the potential bias associated with self-reported measures. However, despite obtaining statistically significant results, it is important to consider that the changes in the VAS were below the thresholds established to be considered minimally clinically relevant changes. Therefore, these results should be interpreted with caution, especially considering that a weak effect size was demonstrated in this context.

Continued research on physical therapy as a treatment for TMDs is imperative due to the low methodological quality of the current physical therapy methods used to address various dimensions of TMDs [22,35]. Additionally, it should be noted that manual treatment has been found to be more effective in the short term than other conservative treatments for TMDs, such as electrotherapy or exercises, even in patients with chronic pain [36,37].

Meta-analyses have been instrumental in identifying the most effective treatments for myofascial or arthrogenous TMDs and have recognized differences in the treatment preferences depending on the specific nature of the disorders. These analyses have also evaluated the feasibility of surgical and invasive procedures as therapeutic alternatives. For arthrogenous TMDs, minimally invasive procedures combined with infiltrations of adjuvant pharmacologic agents have been found to be significantly more effective than conservative approaches to pain reduction and improvements in MMO [38]. However, it is important to note that previous analyses did not differentiate between various physiotherapeutic treatment modalities, such as manual therapy, electrotherapy, or exercise, nor did they specifically explore the potential benefits of different manual therapy techniques. Therefore, more detailed and specific research on physiotherapeutic treatments is necessary to address this knowledge gap and the association of physiotherapy treatment with other modalities, such as occlusal splints, pharmacological therapy, self-care, and behavioral therapies.

In contrast, physical therapy, particularly manual therapy, has been demonstrated to be the most effective treatment for reducing pain in the short term (≤5 months) for myofascial TMDS. Botulinum toxin, on the other hand, has been shown to be more effective in the intermediate term (≥6 months) but with inconsistent results and the long-term side effects remaining to be taken into account [39].

It is important to note, however, that both meta-analyses cited identified significant limitations in the studies included, with their methodological quality ranging from very low to moderate. Therefore, it is important to interpret these findings objectively and with caution, recognizing the need for further research to increase their certainty and obtain robust evidence on treatments for TMDS. It is important to take into consideration that in clinical practice, it is common for patients presenting for consultation, especially in chronic cases, to present with a combination of myofascial and arthrogenous TMDs. Therefore, adopting a comprehensive and multimodal therapeutic approach becomes the most sensible strategy. This involves implementing treatments that are effective and supported by evidence at all stages of the therapeutic process, from the short to the long term. By integrating first-line therapies that address both the myofascial and arthrogenous components of TMDs, the treatment efficacy is maximized, and overall improvement in a patient’s condition is promoted.

In this research, it has been suggested that the MMET does not differ significantly in its effects from previously studied manual therapy interventions. The neurophysiological mechanisms underlying improvements in musculoskeletal disorders at both the spinal and supraspinal levels are likely to be similar considering that pain modulation is an attribute of the nervous system and is conceptualized as the net result of complex neural interactions, where physiological and psychological information is integrated into an individual’s experience of pain [40,41]. However, it is important to note that this research has not comprehensively addressed these mechanisms, which limits the discussion on the uniqueness of the MMET compared to other manual therapy techniques. It is proposed that the relief of PTP in the trapezius muscle by the MMET could be due to modulation of the autonomic nervous system, thereby reducing the stress that is usually manifested in this muscle. As the trapezius muscle responds intensely to stress and head position, using the MMET on the mandible could have influenced its relaxation, indirectly alleviating the tensions reflected in the trapezius muscle through neurophysiological and biomechanical adjustments [42].

This study contributes significantly to the scientific evidence by supporting, through a rigorous design, the efficacy and safety of the MMET as an integral part of multimodal treatment for adults with TMDs (in this case, in the short term). Its ability to coexist with other therapies positions it as a promising technique in this field. A notable finding is the observed effect on the trapezius muscles, suggesting a possible bidirectional relationship with disorders of the cervical region, supported by both the theory of a neuromechanical connection within the trigeminal–cervical complex and the theory of the biomechanics between these segments [43,44]. Such findings underscore the importance of considering the anatomical inter-relationships when approaching the treatment of TMDs, which may have significant implications for treatment planning and execution to improve the clinical outcomes.

### 4.2. Limitations and Future Perspectives

However, it is important to recognize the limitations of this study. Firstly, the sample analyzed had a gender bias, with an over-representation of women. Although the prevalence of temporomandibular disorders (TMDs) in the European population is 9% higher in women than in men, this proportion was not reflected in our study [4]. Therefore, further research is needed to evaluate the efficacy of this technique in a more male-representative sample in order to obtain more generalizable results that are representative of the general population. Another limitation of this study is the choice of the control group, who received a sham suboccipital muscle inhibition technique with no therapeutic intent. Although it acted as a placebo, it was not the ideal comparison for assessing the efficacy of the MMET. A more suitable control would be another established manual therapy technique, which would determine whether the MMET offers advantages over other common interventions. Future studies should consider this option. Another limitation is that only the immediate effects of the MMET were evaluated, which limits the clinical relevance of the results to long-term treatment of chronic temporomandibular disorders. This study did not determine whether its benefits persisted beyond the short term. Future research with medium- and long-term follow-ups is needed to assess the duration of the effects and the possible need for repeated sessions. It is also important to note that patients with arthrogenic and myogenic TMDs were included due to the frequent coexistence of both pathologies, especially in chronic processes. In future research, it would be relevant to perform specific studies for each type of TMD, including both arthrogenic and myogenic cases, to inform clinical practice better. No information about the opportunity for combined therapeutic options was included, particularly occlusal splints and pharmacotherapy, primarily because we faced chronic patients.

Continued research is essential to address the existing knowledge gaps and refine the treatment approaches and study designs for TMDs. Ultimately, this study contributes significantly to the scientific understanding of the treatment of TMDs, supporting the MMET as an effective short-term intervention. In the future, further research is warranted to deepen our understanding of the pathophysiology of TMDs and refine the treatment strategies to improve patients outcomes and quality of life.’

## 5. Conclusions

In conclusion, this study shows that the MMET is a safe and effective treatment for TMD in adults, resulting in significant improvements in pain relief, PTP, MMO, and reduced fear of movement. The findings indicate that the MMET offers immediate benefits without the adverse effects commonly linked to other interventions, making it particularly advantageous for short-term management of TMD. Furthermore, the ability of the MMET to complement other therapeutic approaches enhances its utility within a multimodal treatment framework.

## Figures and Tables

**Table 1:** Means and standard deviations of baseline characteristics of each group and test for normality.

Outcome	EG (*n* = 31)	CG (*n* = 30)	*p*-Value
	$\bar{x}$ ± SD	$\bar{x}$ ± SD	Sig.
Age (Mean, SD)	40.13 ± 10.28	38.47 ± 11.39	0.551
Female, *n* (%)	25 (80.64%)	25 (83.33%)	0.785
Weight (kg)	69.48 ± 15.43	64.93 ± 14.25	0.237
Height (cm)	164.14 ± 8.09	160.18 ± 8.90	0.074
BMI (kg/m^2^)	25.73 ± 5.27	25.25 ± 4.98	0.717
VAS (mean, SD)	5.69 ± 1.95	5.44 ± 2.67	0.681
PTP, Trapezius R (kg/cm^2^)	0.90 ± 0.44	1.03 ± 0.90	0.470
PTP, Upper Trapezius L (kg/cm^2^)	0.92 ± 0.43	1.00 ± 0.70	0.576
PTP, Masseter R (kg/cm^2^)	0.61 ± 0.30	0.63 ± 0.40	0.799
PTP, Masseter L (kg/cm^2^)	0.58 ± 0.28	0.65 ± 0.39	0.438
PTP, External Pterygoid R (kg/cm^2^)	0.66 ± 0.34	0.65 ± 0.40	0.900
PTP, External Pterygoid L (kg/cm^2^)	0.71 ± 0.35	0.72 ± 0.42	0.909
PTP, Digastric Muscle R (kg/cm^2^)	0.55 ± 0.27	0.54± 0.34	0.987
PTP, Digastric Muscle L (kg/cm^2^)	0.53 ± 0.26	0.58 ± 0.36	0.575
ROM, Opening (mm)	38.88 ± 9.08	32.47 ± 8.80	0.007
ROM, Deviation R (mm)	8.56 ± 2.3	7.82 ± 2.93	0.271
ROM, Deviation L (mm)	8.82 ± 2.4	7.46 ± 2.74	0.044
Kinesophobia (mean. SD)	30.32 ± 6.77	31.67 ± 7.19	0.455

Abbreviations: cm: centimeters; kg: kilograms; L: Left; cm: centimeters; mm: millimeters; R: right. Values expressed as means ± standard deviation (SD) and *p*-values (*p*). *p*-value: Shapiro–Wilk test.

**Table 2:** Values of the outcome variables before and after treatment of both groups.

	Experimental Group (*n* = 31)			Control Group (*n* = 30)			*p*. ^c^	*d*
	Pre	Post	*p*. ^a^	Pre	Post	*p*. ^b^		
	$\bar{x}$ ± SD	$\bar{x}$ ± SD	Sig.	$\bar{x}$ ± SD	$\bar{x}$ ± SD	Sig.	Sig.	Sig.
VAS (Mean, SD)	5.69 ± 1.95	5.20 ± 1.81	<0.001	5.44 ± 2.67	5.58 ± 2.59	0.013	<0.001	0.427
PTP, Trapezius R (kg/cm^2^)	0.90 ± 0.44	1.33 ± 0.71	0.025	1.03 ± 0.90	1.04 ± 0.98	0.390	<0.001	0.300
PTP, Upper Trapezius L (kg/cm^2^)	0.92 ± 0.43	1.30 ± 0.70	0.011	1.00 ± 0.70	0.94 ± 0.80	0.047	<0.001	0.322
PTP, Masseter R (kg/cm^2^)	0.61 ± 0.30	0.89 ± 0.45	0.009	0.63 ± 0.40	0.60 ± 0.44	0.087	<0.001	0.184
PTP, Masseter L (kg/cm^2^)	0.58 ± 0.28	0.86 ± 0.44	0.014	0.65 ± 0.39	0.62 ± 0.43	0.086	<0.001	0.228
PTP, External Pterygoid R (kg/cm^2^)	0.66 ± 0.34	0.92 ± 0.52	0.012	0.65 ± 0.40	0.62 ± 0.45	0.075	<0.001	0.234
PTP, External Pterygoid L (kg/cm^2^)	0.71 ± 0.35	0.99 ± 0.64	0.009	0.72 ± 0.42	0.63 ± 0.43	<0.001	<0.001	0.371
PTP, Digastric Muscle R (kg/cm^2^)	0.55 ± 0.27	0.78 ± 0.45	0.017	0.54± 0.34	0.56 ± 0.43	0.296	<0.001	0.215
PTP, Digastric Muscle L (kg/cm^2^)	0.52 ± 0.26	0.78 ± 0.41	0.013	0.58 ± 0.36	0.50 ± 0.41	0.004	<0.001	0.238
ROM, Opening (mm) *	38.77 (34.04–45.83)	50.49 (46.27–51.87)	<0.001	33.39 (23.87–39.25)	32.45 (24.65–37.61)	0.149	<0.001	0.742
ROM, Deviation R (mm)	8.56 ± 2.3	10.35 ± 1.96	<0.001	7.82 ± 2.93	8.09 ± 2.57	0.275	<0.001	0.453
ROM, Deviation L (mm) *	9.19 (6.98–10.22)	11.09 (9.2–12.13)	<0.001	7.15 (5.62–8.86)	7.7 (5.30–9.43)	0.242	<0.001	0.602
Kinesophobia (Mean, SD)	30.32 ± 6.77	23.58 ± 5.81	<0.001	31.67 ± 7.19	30.83 ± 8.35	0.156	<0.001	0.259

Values expressed as means ± standard deviation (SD) and *p*-values (*p*). *p*. ^a^: Student’s *t*-test for dependent samples for the EG. *p*. ^b^: Student’s *t*-test for dependent samples for the CG. *p*. ^c^: intergroup *p*-value (Student’s *t*-test for independent samples). *d*.: Estimation of the size of the intergroup effect (Cohen’s d). *: non-parametric analysis (Wilcoxon’s test for intragroup and Mann–Whitney test for intergroup differences). Expressed as medians and interquartile ranges.

## Data Availability

The data presented in this study are available upon request from the corresponding author. The data are not publicly available due to compliance with data protection regulations.
